# Quantitative Susceptibility Mapping of the Cervical Spinal Cord at 3T and Application to Multiple Sclerosis

**DOI:** 10.1002/nbm.70296

**Published:** 2026-04-28

**Authors:** Benjamin Streichenberger, Mathieu Santin, Ludovic de Rochefort, Quentin Duché, Catherine Guillemot, Samira Mchinda, Stéphane Roche, Anne Kerbrat, Elise Bannier

**Affiliations:** ^1^ EMPENN ERL U1228 Univ Rennes, Inria, CNRS, Inserm, IRISA Rennes France; ^2^ Center For NeuroImaging Research (CENIR) Paris Brain Institute (ICM) Paris France; ^3^ Ventio Marseille France; ^4^ Radiology Department CHU Rennes Rennes France; ^5^ Neurology Department CHU Rennes Rennes France

**Keywords:** fat‐water separation, multiple sclerosis, quantitative susceptibility mapping, spinal cord

## Abstract

Quantitative susceptibility mapping (QSM) is an advanced MRI technique that links phase variations to the local tissue susceptibility. In multiple sclerosis (MS), QSM has shown promise in characterizing brain lesions by assessing chronic inflammation and myelin content. However, in the spinal cord (SC), the question remains as to whether QSM can classify MS lesions as demyenilated/remyenilated and detect chronic inflammation. SC QSM poses novel challenges due to the small size, mobility and curvature of the cord, as well as magnetic field inhomogeneities caused by nearby vertebrae and physiological movement. This study compares two QSM processing methods for the SC. One method uses the IDEAL (iterative decomposition of water and fat with echo asymmetry and least‐squares estimation) algorithm to account for potential fat‐related signal contributions. The second assumes fat has negligible impact. Both approaches employ the PDF (projection into dipole fields) algorithm to remove the background field and the MEDI (morphology enabled dipole inversion) technique for solving the field‐to‐susceptibility inversion. A 2‐sequence MRI protocol was developed to acquire in‐phase (IP) and out‐of‐phase (OOP) QSM data. Eight healthy controls (HC) and twenty MS patients were scanned on a 3T Prisma scanner. We obtained the first high‐resolution axial QSM maps of the cervical SC (C3‐C5), clearly distinguishing gray matter (GM), white matter (WM), and MS lesions. Results showed that accounting for fat using IDEAL did not meaningfully improve the estimation of the total magnetic field or the overall quality of QSM maps. These results open up exciting possibilities for SC susceptibility imaging in MS.

## Graphical abstract

We adapted to the spinal cord (SC) an MRI technique called quantitative susceptibility mapping (QSM) to produce the first high‐resolution axial QSM maps of the cervical SC. Applied to multiple sclerosis, it reveals differences between gray matter, white matter, and lesions, suggesting its potential for improved lesion characterization.

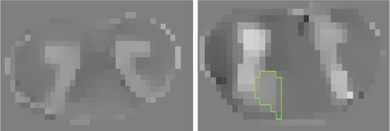

**Abbreviations**
CAL: chronic active lesion
FWS: fat‐water separation
GM: gray matter
GRE: gradient echo
HC: healthy control
IP, in‐phase
MEDI: morphology enabled dipole inversion
MS: multiple sclerosis
OOP: out‐of‐phase
PDF: projection into dipole fields
PRL: paramagnetic rim lesion
QSM: quantitative susceptibility mapping
SC: spinal cord
SCT: spinal cord toolbox
SMV: spherical mean value
WM: white matter

## 1 Introduction

QSM [1, 2] is a promising MRI technique that is part of susceptibility‐based imaging, a field that has become popular in the MS community in recent years because of its ability to assess the presence of chronic inflammation within MS lesions in the brain. QSM is sensitive to diamagnetic components such as myelin and to paramagnetic elements such as iron, making it possible to identify different types of MS lesions, including chronic active lesions (CAL), without relying on contrast enhancement [3]]. CAL are very relevant in MS because they contain chronic inflammation and are associated with disease severity [4]. In brain QSM, CAL correspond to paramagnetic rim lesions (PRLs) [5], which are lesions with iron accumulation in microglia (macrophages) at the lesional edge. These PRLs are highly specific for MS [6] and can serve as a new imaging biomarker in MS monitoring [7].

To date, the potential of QSM to investigate SC lesions and characterize chronic inflammation in the cord of people with MS has not been explored. Yet, SC lesions are seen in up to 80% of people with MS [8] and have a strong prognostic value [9]. A recent histopathologic study [10] found a high prevalence of 41% CAL among SC lesions of 119 MS patients. Therefore, an MRI tool that could quantify the myelin content and inflammatory status of SC MS lesions would be very useful for MS follow‐up.

A great deal of work has gone into developing QSM methods for research purposes, with almost exclusive application to the brain [11, 12]. These different QSM algorithms are largely collected in a common interface called Sepia [13]. However, while QSM is available for applications in the brain, its feasibility and interest have not yet been demonstrated for the SC. This limitation is due to the challenges of SC MRI, including the size and shape of the SC requiring higher resolution, the cardiac and respiratory movements of the patient (implying phase and $R2^{*}$ artifacts), and magnetic field inhomogeneities. Another challenge is the need to adapt the brain QSM reconstruction pipelines to extract SC QSM maps. To the authors' knowledge and as mentioned in [14], high resolution SC QSM applied to MS is not available in the literature and is a novel and challenging problem that we begin to address in this work. Yet, a susceptibility‐weighted imaging sequence at 7T was applied to the cervical SC in nine MS patients in [15]. The authors found some evidence of the presence of central vein sign and PRLs in two thirds of the patients.

QSM applications exist in the human body outside the brain [16]. In particular, QSM of the spine has been applied in a limited number of studies [17, 18] with sagittal orientation and low resolution that would not enable good visualization of SC MS lesions. In these studies, the presence of fat in soft tissues near the SC (especially the vertebrae) prompted the authors to perform a fat water separation (FWS) technique to compute the field map. For the SC, an interesting question is whether or not it is necessary to consider the fat contributions to the MRI signal.

In this study, we propose a 2‐sequence MRI acquisition protocol to obtain SC QSM maps in HC and MS patients, we investigate the influence of fat on SC QSM, and we analyze the susceptibility values and patterns in the SC and in MS lesions.

## 2 Materials and Methods

### 2.1 Prerequisites for SC QSM

QSM in the SC is in principle more challenging than brain QSM because of anatomical factors (small size of the SC) and physiological motion (respiratory and cardiac effects on the MRI signal). Additionally, QSM applications outside the brain may include fat in the signal model [16]. In the brain, most of the signal is generated by protons in water. When a part of the signal is generated by protons in fat, a FWS technique has to be used to compute the field inhomogeneities (or total field), which is needed in QSM to compute the internal field and the final susceptibility map [1, 2]. Indeed, water and fat have shifted resonance frequencies, and this difference is known as the fat‐water chemical shift. It also means that the spins of water and fat go In Phase (IP) and Out of Phase (OOP) with each other as a function of time.

For brain QSM, the temporal signal at the n‐th echo time $TE_{n}$ in a multi‐echo gradient echo (GRE) brain sequence is usually modeled as follows [1]:

(1) $$s(TE_{n})=a(TE_{n})e^{-i\Delta B\omega_{0}TE_{n}}$$

where $a(TE_{n})$ is a complex amplitude and $\omega_{0}$ is the Larmor frequency. To obtain the total field ΔB, a field fitting between the model Equation (1) and the MRI data is performed. Several methods exist to solve this fitting, including phase difference between successive echoes [19] and non‐linear least‐square problem per voxel [1]. In case of FWS, the fat contributions are included in the MRI signal model in the form of a multi‐peak fat model [20]. The temporal signal at the n‐th echo time $TE_{n}$ in a multi‐echo GRE sequence in the presence of a sample component of fat can be expressed as

(2) $$s(TE_{n})=e^{-R2^{*}TE_{n}}\left(\rho_{W}+\rho_{F}\overset{K}{\underset{k=1}{\sum }}\alpha_{k}e^{-i2\pi \Delta f_{k}TE_{n}}\right)e^{-i2\pi \Delta BTE_{n}}$$

where $R2^{*}$ is the apparent transverse relaxation rate, $\rho_{W}$ and $\rho_{F}$ are the water and fat complex signals at time TE = 0, $f_{k}$ is the chemical shift of the k‐th fat peak (k=1,..,K), and, $\alpha_{k}$ is the relative amplitude associated with the k‐th peak such that $\sum_{k=1}^{K}\alpha_{k}$ = 1. The estimation of the total field ΔB on $N_{e}$ successive echoes can be done with the nonlinear FWS approach called $R2^{*}$‐IDEAL [17, 18]:

(3) $$\begin{array}{cc} & \Delta B,R2^{*},\rho_{W},\rho_{F}=\text{argmin}\;\overset{N_{e}}{\underset{n=1}{\sum }}\\ & {\left|\;s(TE_{n})-e^{-R2^{*}TE_{n}}\left(\rho_{W}+\rho_{F}\overset{K}{\underset{k=1}{\sum }}\alpha_{k}e^{-i2\pi \Delta f_{k}TE_{n}}\right)e^{-i2\pi \Delta BTE_{n}}\;\right|}^{2} \end{array}$$

This is a nonconvex problem that is heavily dependent on initialization. For our SC study, auto‐regression on linear operations (ARLO [21]) was chosen to initialize $R2^{*}$. The water and fat complex signals were estimated with the Dixon technique. For the latter, IP and OOP images were acquired on the SC with a specific SC QSM GRE protocol. Finally, the classical initialization for ΔB is the null fieldmap, although other types of initializations exist [17].

Once the field ΔB is obtained with or without FWS (using respectively the model Equations 1 and (2)), the SC QSM follows the same steps as for the brain, that is, removal of the background field and dipole inversion [1, 2].

### 2.2 Acquisition Protocol

MR acquisitions were performed on a Magnetom 3T Prisma running VE11E and a 64 channel head coil with main field $B_{0}=3T$. The MRI protocol for SC QSM was optimized to address the different challenges of SC imaging. Following the recommendations in [16], the repetition time (TR) was taken long enough to collect a large number (> 6) of echoes, which is recommended for robust field fitting, especially for the $R2^{*}$‐IDEAL FWS technique. In addition, the in‐plane resolution was taken high enough to visualize the SC MS lesions, while maintaining a reasonable trade‐off between acquisition time and signal‐to‐noise ratio (SNR).

**FIGURE 1:**
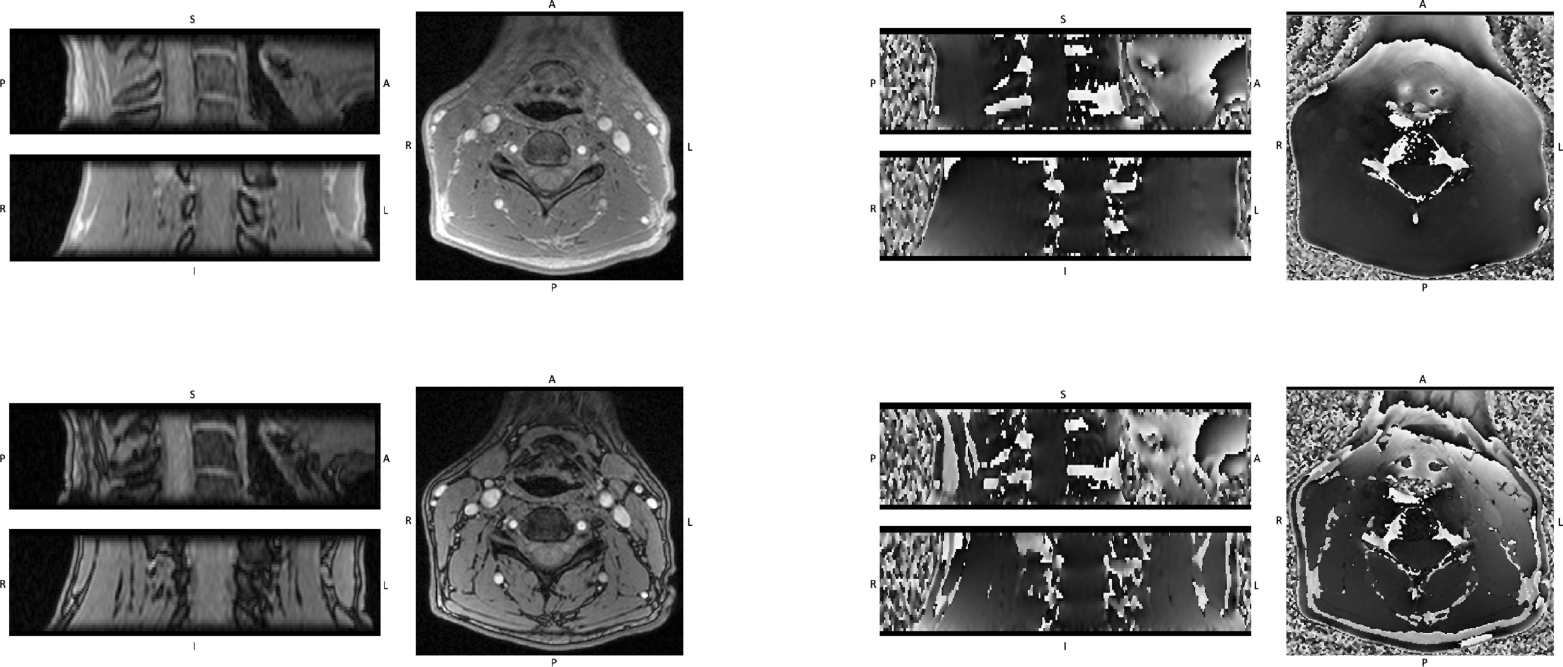
Example of magnitude (left) and phase (right) images of the 1st echoes of IP (top) and OOP (bottom) SC QSM data. For each image: top left = sagittal view; bottom left = coronal view; right = axial view.

In the end, two 3D GRE sequences, respectively with IP and OOP bipolar echoes, were acquired with the following acquisition parameters: TR = 33 ms, 12 echoes each, 0.4×0.4×1 mm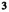 voxel size, 12° flip angle, 1000 Hz/pixel readout bandwith, 154×154 mm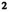 FOV, 36 axial slices, 2 min 30 s acquisition time each. The first sequence corresponds to IP echoes, and only the first echo $TE_{1}$ = 2.64 ms was partially in phase (PIP), because it was not possible to achieve a shorter IP echo of 2.46 ms with our MRI setup. The other IP echo values were $TE_{2}$ = 4.92 ms and ΔTE = 2.46 ms (from the second echo). The second sequence corresponds to OOP echoes with $TE_{1}$ = 3.69 ms and ΔTE = 2.46 ms. The QSM axial acquisition was centered in C4 and extended between C3 and C5 included. Figure 1 shows an example of magnitude and phase images of the first echoes of IP (top) and OOP (bottom) series.

### 2.3 Methodology for Spinal Cord QSM

**FIGURE 2:**
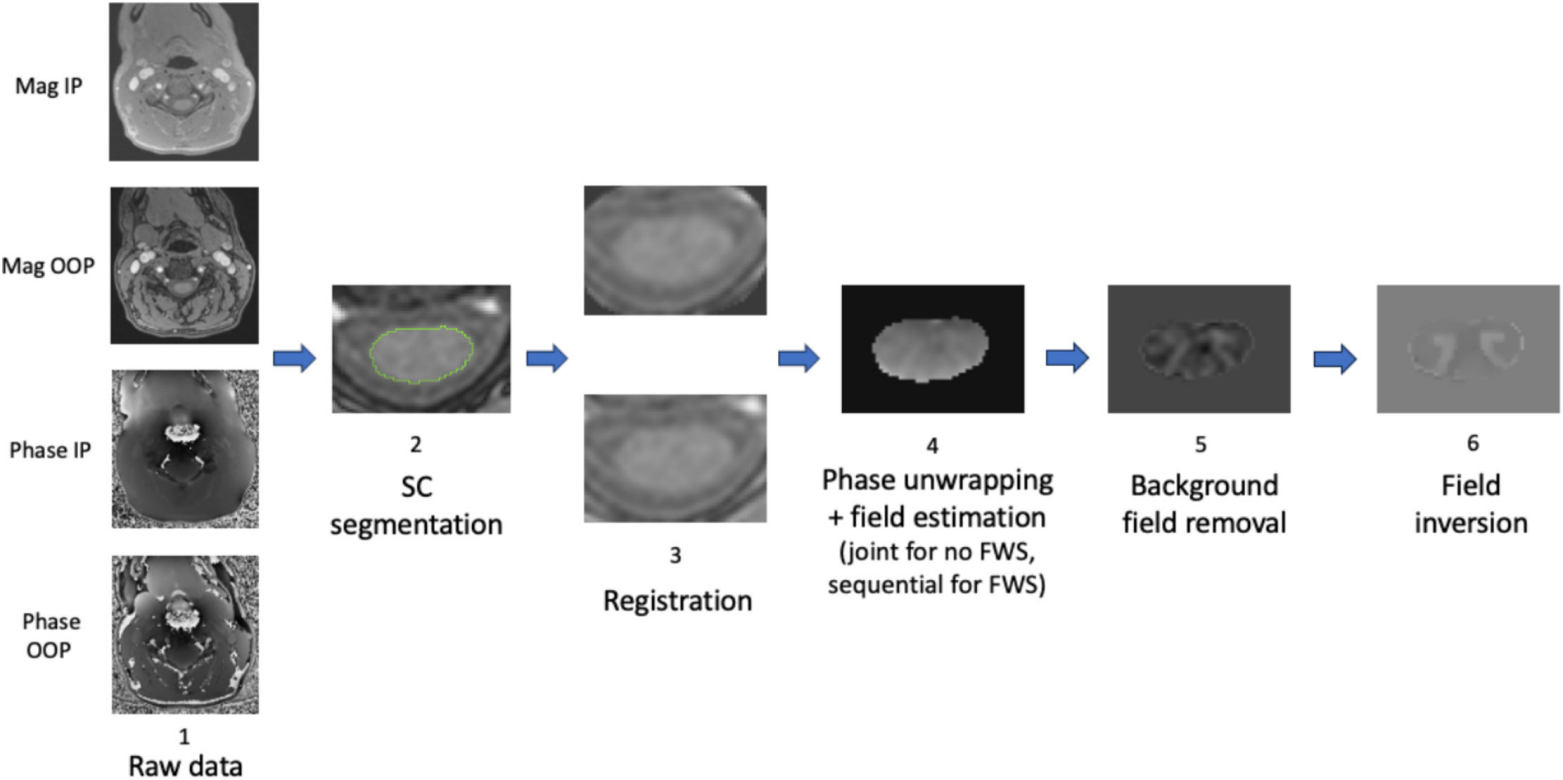
SC QSM processing pipeline. 1: IP and OOP data collection; 2: SC segmentation on GRE OOP magnitude; 3: Registration of IP data to OOP data (the 2 images are IP magnitude of echo 1, the bottom one has been registered and the top one has not), followed by correction of phase offset between echoes for each bipolar IP and OOP acquisition; 4. Phase unwrapping and field estimation. These steps are performed jointly when using the optimum weights algorithm (no FWS) and sequentially when using the $R2^{*}$‐IDEAL model (FWS); 5: Background field removal (PDF); 6: Dipole inversion to obtain the susceptibility map (MEDI). Steps 5 and 6 are applied identically for both FWS and no FWS.

The processing pipeline for SC QSM is presented in Figure 2.

The SC segmentation from step 2 was done on the first echo of the OOP GRE magnitude image with LongiSeg4MS (Longitudinal Segmentation for MS, https://gitlab.inria.fr/amasson/longiseg4ms), an automatic deep‐learning‐based tool for MS lesion detection developed in house. We performed SC segmentation on the first OOP echo because the contrast between the SC and the surrounding tissue was greater than in IP images. We also excluded the top and bottom slices with a lower SNR from the mask. This step had a significant impact on the resulting susceptibility map, as QSM is highly dependent on the choice of masking [22].

The registration of IP data to OOP data of step 3 consists of a linear transformation (translation) followed by a non linear transformation, using the open source software for medical image processing Anima (https://anima.readthedocs.io/en/latest/). The registration function was computed by registering the IP magnitude of echo 1 to the OOP magnitude of echo 1. To avoid undesirable phase effects during registration, the function was applied to the real and imaginary parts of each echo, and the results were then used to reconstruct the registered magnitude and phase images. This registration step was followed by correcting the intra‐acquisition phase offset between echoes [23] for both bipolar acquisitions (IP and OOP). Performing the registration first helps avoid interpolating phase‐corrected data, thus preserving the accuracy of the correction. The effect of performing registration prior to field estimation is illustrated in the Supporting Information (Figures S1 and S2), showing voxel‐wise unwrapped phase evolution with and without prior registration.

For step 4, as described in Section 2.1, the total field ΔB was computed without and with FWS using respectively model Equations (1) and (2). We note that the number of echoes in the two equations is $N_{e}=2N=2\times 12=24$. For the reconstruction without fat contributions, the total field was estimated using the “optimum weights” algorithm implemented in the open‐source software Sepia. This algorithm computes a weighted combination of the phase differences between successive echoes [19]. A 3D Laplacian‐based phase unwrapping [24], based on a wrap‐insensitive Laplacian formulation of the phase [25], is applied to these phase differences, which are then accumulated to reconstruct the unwrapped phase used for linear field fitting. For FWS, the phase was first unwrapped using the same Laplacian‐based method before solving the $R2^{*}$‐IDEAL problem. Following [26], the $R2^{*}$‐IDEAL fitting was weighted using for each echo the SC SNR of the phase image. The latter was computed with the Spinal Cord Toolbox (SCT, version 6.5) [27].

For steps 5 and 6, we chose the combination of PDF and MEDI methods [22, 28, 29]. The $\ell^{2}$ norm of the magnitude images of each IP and OOP echo was used as the anatomical image for the MEDI algorithm. The QSM referencing was done using the global mean susceptibility. In addition, the spherical mean value (SMV) radius parameter was set equal to zero. For brain applications, the SMV is taken to be several times the voxel size. Here, due to the small size of the region of interest, its use would induce an important loss of susceptibility information and contrast, and the anisotropic MRI resolution is also not well suited to SMV. For all other parameters of PDF (tolerance, iterations, pad size) and MEDI (lambda, edge mask threshold), the default values in Sepia were kept.

### 2.4 Quantitative and Statistical Comparison of QSM With and Without FWS

To evaluate the potential influence of fat modeling on SC QSM, we performed qualitative, quantitative and statistical comparisons between QSM maps generated using R2*‐IDEAL (with FWS) and those generated using classical signal modeling without fat contributions (see Equation (1)). Qualitative comparison consisted of visual inspection of the two SC QSM maps for all HC. For the quantitative assessment, we performed linear regressions on susceptibility value of concatenated voxels from all HC, obtained with and without FWS, separately for GM and WM. GM was segmented using the SCT on the $\ell^{2}$ norm of IP/OOP magnitude images. WM was computed as a simple difference between the SC mask and GM segmentation. For statistical analysis, we tested for normality using the Shapiro‐Wilk test, followed by paired *t*‐tests to compare susceptibility values between QSM with and without FWS. A p‐value <0.05 was considered statistically significant.

### 2.5 In Vivo Measurements

After approval from the Institutional Review Board, SC QSM was acquired on a Magnetom Prisma scanner running VE11 on 8 HC and 20 MS patients as part of the VHD study (Very High Definition, substudy of the HD cohort within the French MS registry (OFSEP database), clinical trials ID: NCT05622643). The mean age of MS patients was 41.5 years old (range 28‐65), with 70% of women. Nineteen patients had relapsing‐remitting MS, and one patient had secondary progressive MS. The average disease duration was 14.6 years (range 5–44). Disability, assessed by the Expanded Disability Status Scale (EDSS), averaged 1.675 (range 0–6.5).

MS lesions were automatically segmented on the T2
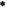
‐weighted multi echo gradient echo (MEDIC) images using the SCT (image resolution: 0.4×0.4×3 mm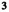). The resulting lesion masks were then manually reviewed and corrected by an experienced neurologist using standard sagittal T2‐weighted turbo spin‐echo (T2w TSE) and 3D UNI MP2RAGE images [30, 31] to confirm lesion presence and remove false positives. Finally, the lesions were registered (with SCT) in the QSM space to visualize QSM patterns and to analyze susceptibility values. Lesions located at the edges of the acquisition volume in C3 and C5, where QSM is more susceptible to artifacts due to lower SNR and reduced algorithm robustness, were excluded from the lesion count. A total of 28 lesions were identified across the 20 patients. Only three patients had no lesions between C3 and C5; seven patients had one lesion, six had two lesions, and three had three. One patient was excluded from the analysis due to noisy raw data.

## 3 Results

### 3.1 SC QSM Maps Obtained in HC and Comparison With and Without FWS

**FIGURE 3:**
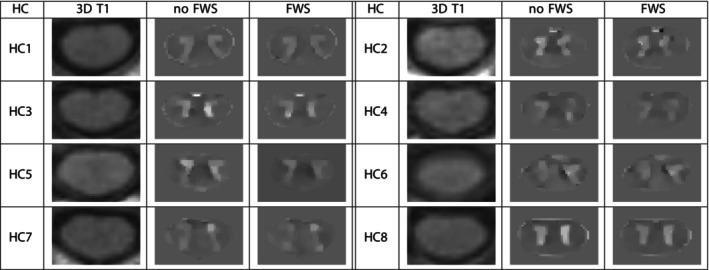
Visual comparison of SC QSM with and without FWS on 8 HC; for each HC, display of a 3D T1w image registered in the QSM space. QSM slices taken in the middle of C4. Susceptibility values between [−30;30] ppb.

We present the QSM maps with and without FWS of the 8 HC in Figure 3. For each HC, three images were displayed: a 3D T1w image registered (with SCT) in the QSM space, a QSM image without FWS, and a QSM image with FWS. The QSM was taken in the middle of the vertebra C4, because the MRI acquisition was centered on C4. Besides, we only had pieces of C3 and C5 (the top and the bottom, respectively), where the SNR is lower and where more errors can occur due to the upper and lower limits [1]. The T1w image was used to help clearly identify the SC boundaries on QSM images. Figure 3 shows that the segmentation of the SC on GRE data, where QSM is computed, is slightly smaller than the actual size of the SC shown by the T1w image. This segmentation helps minimize partial volume effects between CSF and WM in the QSM calculations.

An important point to note in Figure 3 is that SC QSM with and without FWS appear visually very similar for all HC. In both cases, the H‐shape of the GM is distinguishable and hyperintense. The GM of the QSM without FWS seems to have slightly stronger susceptibility values than the QSM with FWS. This results in a more hyperintense signal and a more visible H‐shape. The WM exhibits a relatively iso‐hypointense signal with greater heterogeneity than the GM. QSM with FWS appears to have smoother WM susceptibility values than QSM without FWS (see for example HC2, HC3, HC4 or HC5). One can also see that the QSM maps of HC6 are of lower quality. They have a large heterogeneity in WM susceptibility values and poorly defined GM boundaries. Overall, QSM maps allow for a clear distinction of SC GM and WM, which is promising for the application to MS and for characterizing SC lesions. Additionally, the small visual differences between QSM with and without FWS in our dataset do not seem important for the intended use of SC QSM for MS.

**FIGURE 4:**
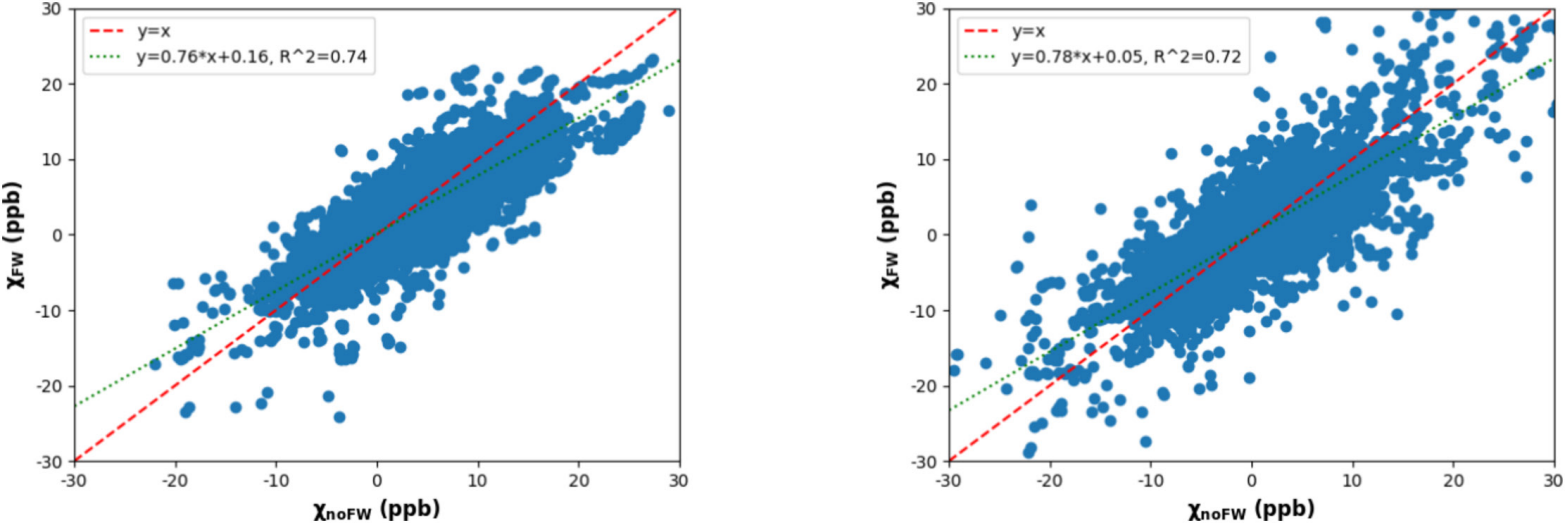
Scatter plots with global linear regression across voxels and HC comparing QSM measurements with and without FWS at vertebra C4, for GM (left curve) and WM (right curve).

Following the qualitative comparison of QSM with and without FWS, we performed a more quantitative comparison with linear regressions on concatenated voxel QSM values at vertebra C4 from all HC, obtained with and without FWS (Figure 4). The left image corresponds to GM data, while the right image corresponds to WM data. Figure 4 illustrates the same trends as Figure 3. There is a strong correlation in both GM and WM cases ($R^{2}=0.74$ and $R^{2}=0.74$, respectively), indicating good consistency between the two QSM methods. The slopes are less than 1 (0.76 and 0.78, respectively), confirming that QSM with FWS tends to slightly underestimate susceptibility compared to QSM without FWS. Finally, the WM distribution is more dispersed, and the variability is slightly higher. This is consistent with visual observations showing greater heterogeneity in WM susceptibility values than in GM values.

Finally, we computed the mean susceptibility values in GM and WM at the C4 vertebral level, averaged over all voxels and all HC. Results are summarized in Table 1. In GM, QSM with FWS yielded slightly lower susceptibility values compared to QSM without FWS. The difference was statistically significant (mean ± SD: 2.3±0.8 ppb vs. 3.1±0.9 ppb, respectively; p=0.0002). In WM, a significant difference was also observed (mean ± SD: −0.6±0.3 vs. −0.7±0.3; p=0.0034), although the absolute difference between the two methods was smaller than in GM. To further evaluate the consistency of QSM values across tissues, we compared susceptibility values between GM and WM in HC at C4. A highly significant difference in susceptibility between GM and WM was observed for both SC QSM approaches (p<0.0001), confirming that both methods preserve expected tissue contrast.

**TABLE 1:** Mean susceptibility values in GM and WM at vertebra C4, averaged over all voxels and all HC, for both SC QSM methods.

Tissue	QSM w/o FWS	QSM with FWS	p‐value*
Gray matter	3.1±0.9 ppb	2.3±0.8 ppb	0.0002
White matter	−0.7±0.3 ppb	−0.6±0.3 ppb	0.0034

* Paired t‐test comparing QSM with and without FWS for each tissue type.

### 3.2 Application of SC QSM to MS Patients

**FIGURE 5:**
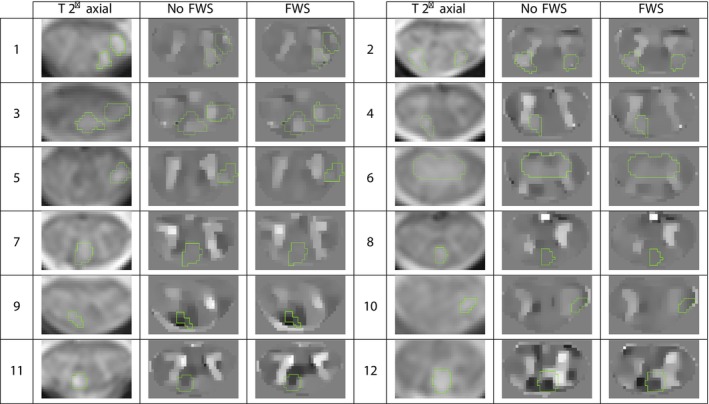
$T2^{*}$ axial MEDIC images and SC QSM with and without FWS for 12 different slices from 9 different MS patients, with a total of 15 lesions located in C4. Susceptibility values between [−30; 30 ppb]. Lesion mask is contoured in green.

SC QSM was applied to a group of 20 people with MS. Our focus was mainly on lesions located in C4, their QSM aspects, and susceptibility values. Figure 5 shows 12 different SC slices (numbered on the figure) from 9 different MS patients, for a total of 15 lesions. For each slice, we present the $T2^{*}$ axial MEDIC images used for lesion segmentation (see Section 2.5) as well as the SC QSM with and without FWS. Lesion mask is contoured in green on the images. Figure 5 shows that different QSM lesion patterns can be observed. Slices 1 to 6 correspond to MS patients with at least one lesion in hypersignal (i.e. a lesion with positive susceptibility values compared to surrounding tissue). Slices 7 and 8 correspond to lesions in isosignal with values close to those of WM. Slices 9 and 10 correspond to lesions in hyposignal (i.e. lesions with strong negative susceptibility values compared to surrounding tissue). Finally, slices 11 and 12 correspond to lesions with a more heterogeneous QSM pattern, which could suggest a pattern similar to the PRL that can be found in the brain.

For slices 1–6 of Figure 5, we observe that the lesions are either completely in hypersignal, as with the lesions in slices 4 and 6, or only part of the lesion is in hypersignal, as with the lesion on the right of slice 1 or the lesion in slice 5. Similar to the average susceptibility value in GM for HC, positive susceptibility values appear slightly lower in the case of QSM with FWS than without (see, for example, the lesions in slice 2 or the lesion in slice 4). Slice 3 also shows that the GM H‐shape is not discernible and that the QSM map is more difficult to interpret. This is actually an MS patient with an important lesion load in the SC, which could result in less GM/WM contrast due to more diffuse SC microstructure damage. Nevertheless, hypersignals can be seen in the lesions. For slices 7 and 8 of Figure 5, we observe lesions in isosignal and strong similarities between the two SC QSM. In slices 9 and 10 lesions appear in hyposignal. In slice 9, it appears that the lesion in hyposignal might be larger than the $T2^{*}$ axial segmentation suggests. In slice 10, the lesion appears to be more in hyposignal for QSM with FWS than without, where it seems that one GM arm confounds with the lesion in QSM without FWS. Finally, the lesion on slice 11 could indicate a PRL, as those observed in the brain, because it has strong negative values at its center and has a paramagnetic rim. The lesion in slice 12 could also suggest a PRL, but the QSM signal varies considerably in the normally appearing WM, especially for QSM without FWS, making the lesion pattern difficult to interpret.

Lesions were classified as hypointense, isointense, or hyperintense based on their mean susceptibility relative to the average WM value in HC, obtained from QSM without FWS (−0.7±0.3 ppb, see Table 1). Lesions with a mean susceptibility greater than −0.7 ppb by more than 1 ppb (i.e., > 0.3 ppb) were considered hyperintense, while those with a mean value lower than −1.7 ppb were considered hypointense. Lesions with a mean susceptibility between −1.7 ppb and 0.3 ppb were classified as isointense. Using a threshold of ±1 ppb relative to the WM reference ensured that only lesions with substantial susceptibility deviations were classified as hyperintense or hypointense, thereby minimizing the risk of misclassification due to noise or minor physiological variation.

The classification of lesions into hypointense, isointense, or hyperintense categories is summarized in Table 2. For SC QSM without FWS, 7% of the lesions were hypointense, 43% were isointense, and 50% were hyperintense. For QSM with FWS, 14% were hypointense, 47% isointense, and 39% hyperintense. Among the 28 MS lesions, only 5 (18%) were assigned to a different category between the two QSM methods (i.e., hypo/iso or iso/hyper), indicating good agreement in lesion classification. The mean susceptibility of hyperintense lesions was similar between QSM methods (mean ± SD: 1.4 ± 0.8 ppb vs. 1.5 ± 1.2 ppb for QSM with and without FWS, respectively), as well as for isointense lesions (−0.4 ± 0.6 ppb vs. −0.4 ± 0.7 ppb). For hypointense lesions, the mean susceptibility was slightly higher with QSM with FWS (−3.2 ± 2.2 ppb) compared to QSM without FWS (−3.6 ± 2.7 ppb). This difference is explained by the fact that the proportion of hypointense lesions doubled with FWS. A paired t‐test on all 28 lesions confirmed a statistically significant difference (p=0.0006) between the two SC QSM methods, with slightly lower susceptibility values observed with FWS (mean ± SD: −0.1 ± 1.8 ppb vs. 0.3 ± 1.8 ppb). This is consistent with the observation that, in HC, QSM with FWS tended to underestimate susceptibility compared to QSM without FWS.

**TABLE 2:** Distribution and mean susceptibility values of MS lesions across categories, for SC QSM with and without FWS.

	QSM w/o FWS		QSM with FWS		
Type of MS lesions	Percentage	Mean	Percentage	Mean	p‐value*
Hypointense	7%	−3.6±2.7 ppb	14%	−3.2±2.2 ppb	
Isointense	43%	−0.4±0.7 ppb	47%	−0.4±0.6 ppb	
Hyperintense	50%	1.5±1.2 ppb	39%	1.4±0.8 ppb	
All lesions	—	0.3±1.8 ppb	—	−0.1±1.8 ppb	0.0006

* Paired t‐test on susceptibility values across all 28 lesions for both QSM methods.

## 4 Discussion

The aim of this study was threefold: to adapt the QSM technique to the cervical SC; to evaluate the influence of fat on SC QSM; and to establish whether WM, GM, and MS lesions can be distinguished based on QSM signals. Two SC QSM pipelines, in conjunction with a new acquisition protocol configured to recover a cycle of IP/OOP $T2^{*}$‐weighted data, were used to extract SC QSM maps. One pipeline employed linear fitting without FWS to compute the field map, while the other used the FWS $R2^{*}$‐IDEAL algorithm. Both SC QSM then used the PDF method to generate the internal field and the MEDI method for field‐to‐susceptibility inversion. To increase the chances of detecting variations in the QSM signal, especially in MS lesions, a high spatial resolution in the axial plane was chosen.

Visually, the two SC QSM maps, with and without FWS, appeared quite similar across all HC (see Figure 3). The QSM maps showed good contrast, with clear differentiation between WM and GM. Quantitatively, we observed strong correlations between the two pipelines for both GM and WM (see Figure 4), although QSM with FWS tended to slightly underestimate susceptibility values compared to QSM without FWS. At vertebral level C4, the average susceptibility values were approximately one order of magnitude lower than typical QSM values reported in the brain. While brain WM and GM typically exhibit QSM values ranging from −90 to 0 ppb and +30 to +130 ppb, respectively [32, 33], our SC measurements showed average values around −1 ppb for WM and +3 ppb for GM. This difference likely reflects the lower iron content and distinct microstructural properties of SC tissue compared to brain.

For MS patients, demonstrating QSM signal variations within SC lesions was a crucial motivation for developing the SC QSM technique. Our quantitative results (Table 2) show that, for both QSM processing methods (with and without FWS, respectively), the majority of SC lesions were either isointense (47% vs. 43%) or hyperintense (39% vs. 50%), with a smaller proportion of hypointense lesions (14% vs. 7%). These findings are consistent with brain MS lesion QSM distribution reported in the literature. For example in [5] the authors classified over 1600 brain MS lesions either as hyperintense (more than 50%), isointense (around 30%), PRLs (around 13%) and hypointense (around 5%). Regarding susceptibility values, we found that hyperintense SC lesions had mean values around +1.4 to +1.5 ppb, isointense lesions near −0.4 ppb, and hypointense lesions approximately −3.2 to −3.6 ppb. These values are roughly one order of magnitude lower than typical values reported for brain MS lesions [5], consistent with the general trend observed in healthy tissue where SC QSM values are lower than in the brain. Postmortem analyzes showed that these differences in brain QSM signal are linked with different microstructure contents within brain MS lesions: Remyelinated lesions corresponded to hypointense/isointense lesions, chronic inactive lesions to hyperintense lesions, and CAL to PRLs [5]. This highlights the need for future correlation studies between SC QSM and anatomopathology. The development of high resolution QSM for the cervical SC is a first step towards identifying chronic SC inflammation in MS patients. Combined with existing biomarkers of chronic inflammation in the brain, this could be very useful in monitoring MS patients.

The following attempts to compile an exhaustive list of the main limitations of this preliminary study for SC QSM. The first important limitation of our setup is the current acquisition protocol, which comprises two sequences to recover IP and OOP GRE data, respectively. Performing these sequences one after the other is time‐consuming and may result in a slight displacement of the SC between the IP and OOP images due to patient motion between scans, requiring a registration step between IP and OOP data before QSM calculation (see Figure 2). Thus, an inherent registration error exists in this operation.

Initially, the use of two successive acquisitions was due to gradient limitation preventing a short enough delay between IP and OOP echoes. Obtaining IP and OOP data enabled the initialization of the $R2^{*}$‐IDEAL algorithm, even though it was proposed to process without [18]. However, as discussed, accounting for fat in the field fitting did not clearly improve SC QSM quality, despite quantitative differences. If a guideline had to be provided, we would recommend using SC QSM without FWS because it greatly simplifies the post‐processing without any noticeable deterioration of the QSM map. Still, 2 sequences of echoes, that overlap and allow the echo time ΔTE to be divided by 2, had to be kept, even without FWS, because they effectively double the total number of echoes acquired, thereby increasing the amount of data available for field fitting. This increase in echoes is critical, as it leads to a higher overall SNR and more robust estimation of the local magnetic field. This is particularly important in the SC, where small anatomical structures and subtle susceptibility variations make QSM highly sensitive to noise and phase‐fitting errors. In contrast, brain QSM can often achieve reliable results with a few echoes due to larger field variations. We compared QSM reconstructions using the full 24‐echo dataset versus a reduced 12‐echo subset, both without and with FWS (see Supporting Information, Figures S3 and S4). Using fewer echoes led to higher noise, lower tissue contrast, and reduced lesion conspicuity regardless of FWS, confirming that echo number is critical for robust SC QSM estimation. Using an MRI sequence capable of acquiring more echoes in a single run could potentially replace the two‐sequence approach, but should not compromise spatial resolution. Another option would be to perform a 2nd identical repetition and average the data to increase the SNR. Further protocol optimization remains an important avenue for future work.

Another limitation is the choice of algorithms and methods used to feed the SC QSM pipeline, which was based on experience. A real investigative study, such as that conducted in [34] for the brain, is required to determine the most effective algorithms for computing the SC the field map, internal field and final QSM maps. In particular, it would be interesting to adapt to the SC the background field removal algorithms [28], as this could improve the final QSM map. In addition, although our study focused on in vivo data, future work could benefit from the use of numerical or physical phantoms specifically designed for SC imaging [17, 35], which would provide ground truth references to better quantify the accuracy and limitations of SC QSM reconstructions.

It is also important to remember that this study only covers a small part of the cervical SC, in this case the area from the bottom of C3 to the top of C5, which is equivalent to around two vertebrae. The total acquisition time is 5 min (2 min 30 s for the IP sequence and 2 min 30 s for the OOP sequence), so that a QSM acquisition of the entire SC, where MS lesions are present and need to be characterized, would certainly take too long. Also, this study has a limited number of HC and MS patients. A much larger cohort of MS patients must be considered in future studies to generalize and verify the results. Another important future perspective of work is the reproducibility of SC QSM, as it has been done for brain QSM [33, 36, 37]. Finally, we would like to point out that the lesion mask is done on the $T2^{*}$ axial image and then registered to the QSM space. These 2 sequences have different resolutions in the z‐direction (3 mm for $T2^{*}$ axial versus 1 mm for QSM). This means that one slice in the $T2^{*}$ axial image corresponds to 3 or 4 slices in QSM (it is not the same origin). All of this induces a non‐negligible segmentation error that may deteriorate the characterization of lesions in the SC QSM maps.

## 5 Conclusions

In this study, we developed for the first time high‐resolution axial QSM of the cervical SC at 3T, with a new scheme of acquisition protocol that provides an alternance of IP and OOP GRE data for a total of 24 echoes. The influence of fat was evaluated and it was concluded that SC QSM should be used without FWS to simplify the process and because there was no notable drop in QSM map quality. SC QSM was applied succesfully to both HC and MS patients. Distinctions were made between WM and GM, and variations in the QSM signal within the MS lesions were detected. The results of our study may open the way for future research into susceptibility‐based MRI of the SC for MS, with the medium‐term aim of using MRI to identify chronic inflammation in the SC.

## Author Contributions

B. Streichenberger led the analysis and writing of the paper. E. Bannier, M. Santin, L. de Rochefort, S. Roche, and A. Kerbrat led the conceptualization and supervision of the project. E. Bannier, A. Kerbrat, and M. Santin acquired the fundings to support the project. E. Bannier, C. Guillemot, and A. Kerbrat collected the data. Q. Duché and B. Streichenberger were responsible for the data curation and processing. B. Streichenberger, L. de Rochefort, M. Santin, E. Bannier, S. Roche, and S. Mchinda developed the methodology. Finally, all authors contributed to the writing review and editing.

## Funding

This work was supported by RHU PRIMUS, FLI RE4 (QSM‐SPICO), France Sclérose en Plaques (Foundation to support research into MS), and Fondation de l'Avenir (N°AP‐RM‐24‐008). MRI data acquisition was performed at the Neurinfo MRI research facility from the University of Rennes I, University Hospital of Rennes, Inria, CNRS and the Rennes Cancer Center. Neurinfo is also supported by the Brittany Council, Rennes Metropole and GIS IBISA. Ventio received support by the Region Sud and by the French government as part of the France 2030 Plan.

## Conflicts of Interest

The authors declare no conflicts of interest.

## Supporting information



SupMat.pdf

## Data Availability

The data can be obtained upon request to the OFSEP Scientific Board (https://www.ofsep.org/en/data‐access).
