# Supplementary material for: Quantitative Susceptibility Mapping of the Cervical Spinal Cord at 3T and Application to Multiple Sclerosis
Source: NMR Biomed. 2026 Apr 28;39(6):e70296. doi: 10.1002/nbm.70296 (PMC13122293; doi:10.1002/nbm.70296)
Supplement: Supplementary file 1 — SupMat.pdf [file NBM-39-e70296-s001.pdf]

Supplementary Material

Manuscript: Quantitative susceptibility mapping of the cervical spinal cord at 3T and application to multiple sclerosis
Authors: Benjamin Streichenberger et al.

1. Supplementary Methods

1.1 Voxel-wise Phase Evolution
Evolution of the unwrapped phase versus echo time was evaluated for two example voxels. 3D Laplacian unwrapping was applied concurrently with the optimum weight field estimation algorithm using the Sepia framework, as described in the main manuscript. A comparison of phase evolution was performed before and after registering the IP echoes onto the OOP echoes to evaluate the benefits of registration.

1. 2 Impact of Echo Number on SC QSM Reconstruction
To evaluate the impact of the number of echoes on SC QSM reconstruction, the QSM map was computed using either the full 24-echo dataset (12 IP + 12 OOP) or a reduced 12-echo subset. Reconstructions were performed both without FWS and with FWS using the R2*-IDEAL algorithm, following the same processing workflow described in the main manuscript. For SC QSM without FWS, one IP sequence of 12 echoes was used. For SC QSM with FWS, the first 6 IP and 6 OOP echoes were combined to obtain a dataset of 12 echoes.

2. Supplementary Results

1.1 Voxel-wise Phase Evolution
Representative examples of voxel-wise phase evolution for IP and OOP echoes were evaluated in two example voxels (see Fig. S1 and S2). Comparisons before and after registering the IP echoes onto the OOP echoes illustrate the effect of registration on phase consistency across acquisitions. These observations support the use of a two-acquisition protocol (IP + OOP), as combining information from both sequences preserves phase consistency and enhances the robustness of subsequent field estimation.

1. 2 Impact of Echo Number on SC QSM Reconstruction
The effect of using a reduced number of echoes on SC QSM reconstruction was assessed for both reconstructions with and without FWS. Compared to the full 24-echo dataset, the reduced 12-echo reconstructions showed differences in noise level, tissue contrast, and lesion conspicuity (see Fig. S3 and S4.). This effect was consistent across both FWS and non-FWS reconstructions, highlighting that the number of echoes is a critical factor for reliable SC QSM estimation.

3. Supplementary Figures

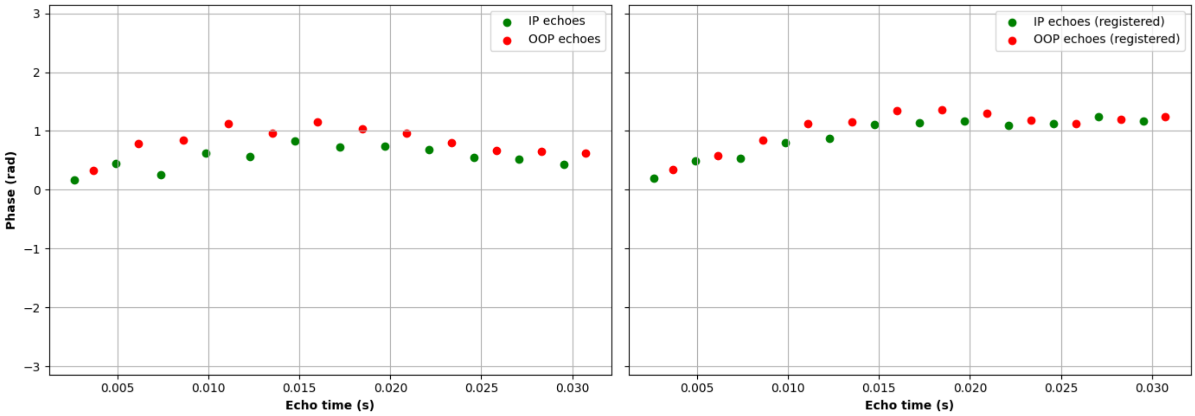

Figure S1: Unwrapped phase with respect to echo time for IP and OOP acquisitions for a MS patient in a WM voxel. Left images correspond to the unwrapped phase before registration. Right images correspond to the unwrapped phase after registration.

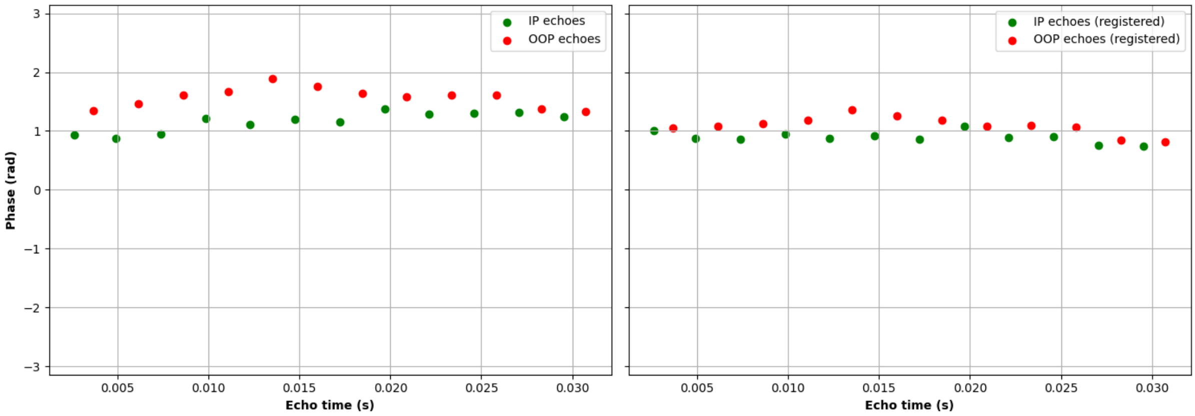

Figure S2: Unwrapped phase with respect to echo time for IP and OOP acquisitions for another MS patient in a GM voxel. Left images correspond to the unwrapped phase before registration. Right images correspond to the unwrapped phase after registration.

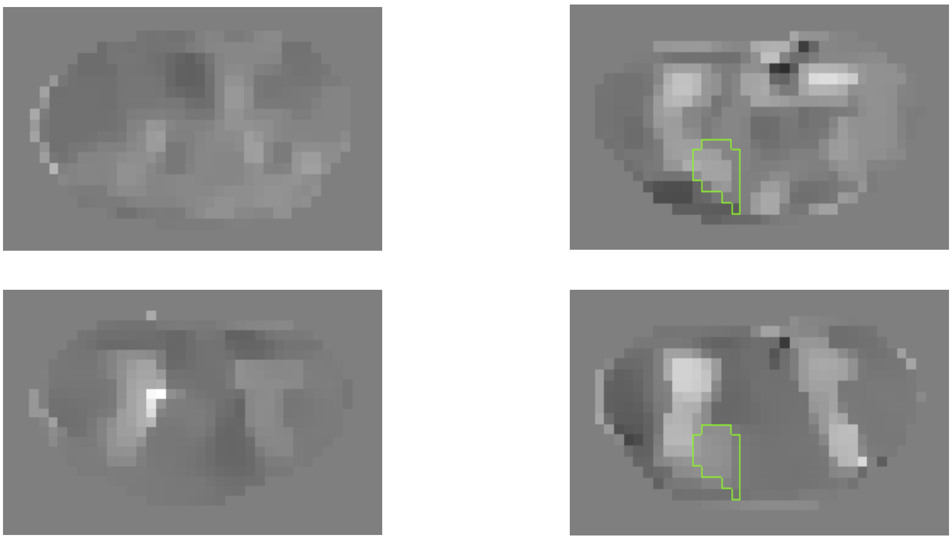

Figure S3: QSM maps of a HC (left) and an MS patient (right) reconstructed without FWS. Top row: reconstruction using a single IP sequence with 12 echoes. Bottom row: reconstruction using the two-sequence protocol.

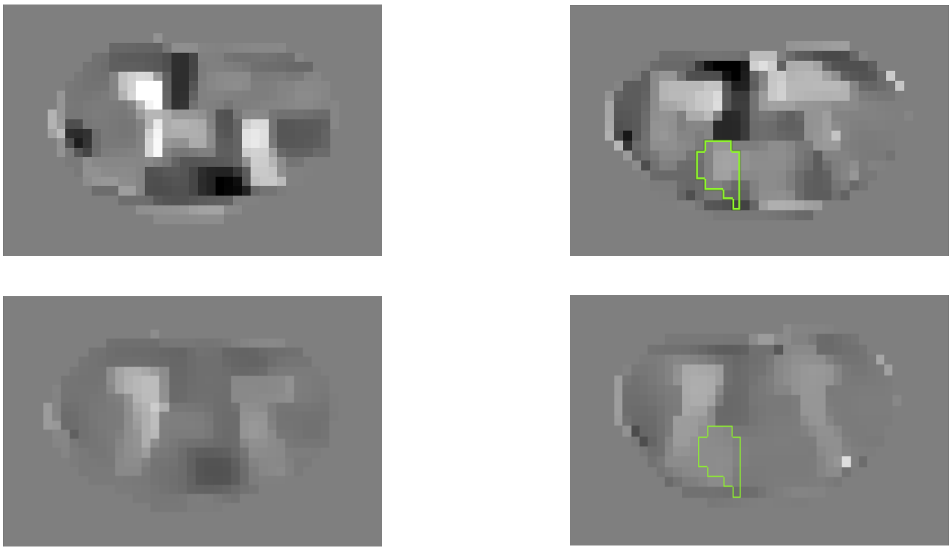

Figure S4: QSM maps of a HC (left) and an MS patient (right) reconstructed with FWS. Top row: reconstruction using the 12 first echoes (6 IP and 6 OOP). Bottom row: reconstruction using the two-sequence protocol.
